# Enhanced Lymphatic Delivery of Methotrexate Using W/O/W Nanoemulsion: In Vitro Characterization and Pharmacokinetic Study

**DOI:** 10.3390/pharmaceutics12100978

**Published:** 2020-10-16

**Authors:** Ji-Hun Jang, Seung-Hyun Jeong, Yong-Bok Lee

**Affiliations:** College of Pharmacy, Chonnam National University, 77 Yongbong-ro, Buk-Gu, Gwangju 61186, Korea; jangji0121@naver.com (J.-H.J.); rhdqn95@naver.com (S.-H.J.)

**Keywords:** methotrexate, nanoemulsion, lymphatic delivery, pharmacokinetics

## Abstract

Methotrexate, which is widely used in the treatment of cancer and immune-related diseases, has limitations in use because of its low bioavailability, short half-life, and tissue toxicity. Thus, in this study, a nano-sized water-in-oil-in-water (W/O/W) double emulsion containing methotrexate was prepared to enhance its lymphatic delivery and bioavailability. Based on the results from solubility testing and a pseudo-ternary diagram study, olive oil as the oil, Labrasol as a surfactant, and ethanol as a co-surfactant, were selected as the optimal components for the nanoemulsion. The prepared nanoemulsion was evaluated for size, zeta potential, encapsulation efficiency, pH, morphology, and in vitro release profiles. Furthermore, pharmacokinetics and lymphatic targeting efficiency were assessed after oral and intravenous administration of methotrexate-loaded nanoemulsion to rats. Mean droplet size, zeta potential, encapsulation efficiency, and pH of formulated nanoemulsion were 173.77 ± 5.76 nm, −35.63 ± 0.78 mV, 90.37 ± 0.96%, and 4.07 ± 0.03, respectively. In vitro release profile of the formulation indicated a higher dissolution and faster rate of methotrexate than that of free drug. The prepared nanoemulsion showed significant increases in maximum plasma concentration, area under the plasma concentration-time curve, half-life, oral bioavailability, and lymphatic targeting efficiency in both oral and intravenous administration. Therefore, our research proposes a methotrexate-loaded nanoemulsion as a good candidate for enhancing targeted lymphatic delivery of methotrexate.

## 1. Introduction

Interest has been steady in the lymphatic system as a pathway for lipid absorption, cancer metastasis, and immune system response. The system has an important role in tissue fluid balance, intestinal absorption of lipid, and immunological functions [1,2]. In addition to its role in normal physiology, the lymphatic system is known to be involved in cancer progression because cancer cells can spread to lymph nodes through lymphatic vessels [3,4]. The lymphatic system has also attracted attention for potentially suppressing cancer metastasis and effectively treating immune-related diseases. As tumor-induced lymphatic remodeling, lymphatic vessel contraction around cancer cells occurs, thereby promoting the spread of cancer cells through altered lymph flow [4]. Therefore, this suggests that lymphatic delivery of anticancer drugs is very important for effective metastatic cancer treatment. Increasing the lymphatic delivery of anticancer drugs and immune-related drugs could improve the therapeutic effects while reducing side effects; in addition, the therapeutic effects might be possible with low doses. In this regard, lymphatic targeting and drug delivery using formulation of anticancer and immune-related drugs could be of great interest for cancer and immune disease treatment.

In a recent report [5], investigators proposed that a lipid coating on the surface of cancer cells in the lymphatic system was a major cause of higher survival and metastasis of cancer cells that were injected into the lymphatic system than were the ones injected into the blood. That study’s findings are further strong evidence that the lymphatic system is a major pathway for cancer metastasis. These findings also suggest that studies on lymphatic targeting and migration of anticancer drugs are crucial, and, in fact, researchers have studied a number of targeting strategies and benefits based on the structural and physiological characteristics of the lymphatic system related to drug delivery [6,7,8,9]. Researchers have also reported on improving bioavailability and efficacy through formulations of conventional drugs [10,11,12,13]. Formulation with polyaminoacid nanocapsule has been attempted to improve lymphatic delivery of docetaxel as an anticancer drug [10]. In addition to anticancer drugs, there were several studies to formulate nanostructured lipid carriers [11], solid lipid nanoparticles [12], and self-microemulsifying drug delivery systems [13] to improve lymphatic delivery for mebendazole, quetiapine, and saquinavir, respectively.

Methotrexate, a folic acid antagonist, has frequently been used as an anticancer and immune-related drug for treating various tumors and autoimmune and inflammatory diseases [14,15]. However, methotrexate has limitations in use because of its low oral bioavailability, short half-life, and severe adverse effects [16,17]. The major side effects of methotrexate have been reported as hepatotoxicity, nephrotoxicity, neurotoxicity, and pulmonary toxicity [16]. To increase the therapeutic effects while resolving these limitations, improvement of drug delivery system through the formulation development of methotrexate has been required. Toward this end, researchers have reported on various methotrexate formulations including micelles [18,19], microsphere [20], nanoparticles [21,22], liposomes [23], and polymersome [24]. In fact, in our previous study [25], we encapsulated methotrexate in nanoparticles composed of poly(lactic-co-glycolic acid) and evaluated its in vivo distribution and lymphatic delivery and we did observe improved lymphatic transport and bioavailability and longer half-life than with free drug.

Formulations using polymers have mechanical stability because of a hardened surface and a solid core, but their structural rigidity makes these formulations less flexible, which may limit penetration into the vascular wall [8,26]. In addition, these hard particles may trigger more phagocytosis by phagocytic elements, and they may cause pain or allergic reactions when administered [27]. In contrast, formulations with a fluid surface, including emulsions, flow more easily through the blood vessels and into the lymph nodes [8]. Nano-sized emulsions are at a sufficiently small scale to penetrate into the lymph, and they have a larger surface area that provides greater absorption [28]. Although there is a concern about instability in the fluid system as a disadvantage of nanoemulsions, it can be sufficiently stabilized through the selection of appropriate emulsifiers (surfactants, co-surfactants, etc.) and an optimized ratio between components [29]. In addition, formulations can be stabilized by using the proper emulsion type. In addition to conventional single emulsions (oil-in-water or water-in-oil), multiple emulsions such as water-in-oil-in-water (W/O/W) are attracting attention as a potentially more stable drug delivery system [30]. W/O/W double emulsions consist of water-in-oil emulsions dispersed in a second continuous water phase. Compared with single emulsions, double emulsions can better protect the encapsulated compounds and resist degradation by the external environment [31,32]. Therefore, in this study, we considered W/O/W nanoemulsion an appropriate formulation for improving lymphatic delivery and bioavailability of methotrexate.

To the best of our knowledge, no researchers have evaluated lymphatic migration and distribution in the body for nanoemulsion containing methotrexate. Nanoemulsification could increase therapeutic effects owing to the improved bioavailability and delivery to the target tissue. In addition, nanoparticles with a hard surface (prepared in our previous report [25]) and nanoemulsions with a soft structure have differences in composition and physicochemical properties even though their nanoscale properties are similar. Accordingly, the two formulations are considered to differ in pharmacokinetic profiles and distribution in the body.

Therefore, for this study, a formulation of methotrexate-loaded nanoemulsions was developed and its in vivo distribution and pharmacokinetic characteristics were evaluated. In particular, lymphatic delivery of methotrexate-loaded nanoemulsion is an interesting topic that has not been previously reported. As mentioned above, to overcome the limitations of methotrexate, it was necessary to study different formulations for improved targeted delivery. To maximize the therapeutic efficacy of methotrexate and reduce side effects. In this regard, it is crucial to evaluate the lymphatic delivery of the nanoemulsion formulation. Based on this in vivo pharmacokinetic study of methotrexate nanoemulsions, we expect to expand research into therapeutic effects, toxicity, and safety of nanoemulsions in the future.

## 2. Materials and Methods

### 2.1. Materials and Reagents

Methotrexate (purity ≥ 99%), phenacetin (purity ≥ 99%), castor oil, corn oil, mineral oil, olive oil, soybean oil, Cremophor^®^ EL, Span^®^ 80, Span^®^ 85, Tween^®^ 80, phosphate buffered saline (PBS), and hydrochloride (HCl) were purchased from Sigma-Aldrich (St. Louis, MO, USA). Labrasol^®^ was supplied by Daejung Chemicals & Metal Co., Ltd. (Siheung-si, Gyeonggi-do, Republic of Korea). Ethanol and sodium chloride (NaCl) were purchased from Duksan (Ansan-si, Gyeonggi-do, Republic of Korea). LC-MS grade methanol, acetonitrile, water (18.2 mΩ), and HPLC-grade ethyl acetate were obtained from Fisher Scientific (Fair Lawn, NJ, USA). LC-MS grade formic acid was supplied by Tokyo Chemical Industry (Tokyo, Japan). All other reagents used for the experiments were in analytical grades.

### 2.2. Screening of Solubility

The solubility of various oils (castor oil, corn oil, mineral oil, olive oil, and soybean oil) and surfactants (Cremophor EL, Labrasol, Span 80, Span 85, and Tween 80) for methotrexate was evaluated to determine the suitable components for nanoemulsion containing methotrexate. Excess amounts (5 mg) of methotrexate were placed in capped vials with 1 mL of each oil or surfactant. Then, the mixture was vortex-mixed and kept in a shaking (70 opm) water bath at 25 °C for 72 h. After reaching equilibrium, the samples were centrifuged at 10,000× *g* for 10 min. The supernatant was taken and diluted with methanol to quantify the methotrexate; concentrations were determined by UPLC-MS/MS (Shimadzu Corp., Kyoto, Japan). The UPLC-MS/MS method used here is the same as the method in our previous study [25]. Brief analysis conditions are described in Section 2.6.2. Quantification of Methotrexate in Biological Samples. As a co-surfactant, ethanol was fixed without a separate solubility test by referring to previously reported studies [33,34,35]. In other words, optimization through solubility screening of oil and surfactant for methotrexate was the main focus for the preparation of methotrexate-loaded nanoemulsion in this study, and co-surfactant, which plays a relatively auxiliary role, was selected by referring to the previous studies.

### 2.3. Constructing Pseudo-Ternary Phase Diagrams

We developed a pseudo-ternary phase diagram to determine the optimal composition and component ratio for the nanoemulsion. We used water titration to construct the diagrams [36]. Based on the results of the solubility screening tests (at 25 °C in water bath), the selected oil was mixed with surfactant or a surfactant/co-surfactant mixture (S_mix_) at ratios ranging from 1:9 to 9:1 (*v*/*v*). Herein, S_mix_ was made with different ratios (*v*/*v*) of 0.5:1, 1:1, 2:1, 4:1, and 1:0 (100% surfactant). Each mixture was titrated dropwise with deionized water (DIW) under gentle stirring. By visual inspection, we determined the time point at which the mixture became turbid to be the end point of titration.

### 2.4. Preparing the Nanoemulsion

Based on the obtained pseudo-ternary diagrams, we prepared W/O/W double nanoemulsion using olive oil, Labrasol and ethanol as oil, surfactant, and co-surfactant, respectively. First, methotrexate was mixed in oil at a ratio of 1:2 (*w*/*v*). Next, to prepare W/O emulsion, the mixture of oil and S_mix_ was titrated with DIW at a rate of 1 mL/min under stirring at 500 rpm with a magnetic stirrer (GCMS-G, Global Lab, Siheung-si, Gyeonggi-do, Republic of Korea). Then, the obtained mixture was stirred at 24,000 rpm for 5 min by high speed homogenization (PT-3100, Kinematica AG, Luzern, Switzerland) in an ice bath; we performed this homogenization process a total of three times. Afterward, a fine W/O nanoemulsion was obtained using a microfluidizer (LV1, Microfluidics, Westwood, MA, USA) for three passes. W/O/W double emulsion was prepared by mixing the obtained W/O emulsion and the external water phase with homogenization at 10,000 rpm for 10 min. The preparation conditions of methotrexate-loaded nanoemulsion were optimized through the following tests: (1) surfactant/co-surfactant ratio (*v*/*v*) in water-in-oil (W_1_/O) emulsion; (2) volume ratio (*v*/*v*) of internal water phase to oil phase (W_1_:O), and W_1_/O emulsion phase to external water phase to external water phase (W_1_/O:W_2_); (3) Ratio (*w*/*v*) of methotrexate:oil in nanoemulsion; (4) number of microfluidizer passes.

### 2.5. In Vitro Characterization of Nanoemulsion

#### 2.5.1. Droplet Size, Zeta Potential, and pH

We assessed droplet size by dynamic light scattering (DLS; SZ-100, Horiba Scientific, Kyoto, Japan), and we measured zeta potential of nanoemulsion using a zeta potential analyzer (SZ-100, Horiba Scientific, Kyoto, Japan). The prepared nanoemulsion was diluted with DIW (1/20, *w*/*v*) and gently mixed for 1 min. The samples were loaded on the measurement cells maintained at 25 °C. The pH of prepared formulation was determined using a pH meter (SevenEasy, Mettler-Toledo, Zürich, Switzerland). We performed these preparation procedures twice more and evaluated the physicochemical properties through the same process as above.

#### 2.5.2. Drug Encapsulation Efficiency

To determine the drug encapsulation efficiency (EE) in nanoemulsion, the formulation was centrifuged at 13,500× *g* for 15 min. We collected unencapsulated methotrexate from the supernatant. Then, the supernatant was diluted with methanol to quantify the amount of unencapsulated drug by UPLC-MS/MS [25]. The analysis conditions are briefly introduced in Section 2.6.2. Quantification of Methotrexate in Biological Samples. We calculated EE as follows:

$$\mathrm{EE}\;(\%)\;=\;\frac{amount\;of\;methotrexate\;in\;formulation-amount\;of\;methotrexate\;in\;supernatant}{amount\;of\;methotrexate\;in\;formulation}\;\times 100$$

#### 2.5.3. Stability Study

We tested the stability of the methotrexate-loaded nanoemulsion through measuring changes in its physicochemical properties (droplet size, zeta potential, EE, and pH) over time. The samples were stored at room temperature (25 ± 1 °C). The analyses were conducted 1, 3, 7, and 14 days after preparation. The 14-day period set in this study was a period in which stability was sufficiently guaranteed to carry out in vitro and in vivo studies after preparing formulation. Accordingly, the stability of the formulation for a short period up to 14 days was confirmed.

#### 2.5.4. Morphological Analysis

We examined the morphological features of methotrexate-loaded nanoemulsion using field-emission transmission electron microscope (FE-TEM; JEM-2100F, JEOL Ltd., Tokyo, Japan). Before observation, the samples were placed on a carbon-coated nickel grid and dried at 25 °C. The samples were observed at an acceleration voltage of 200 kV.

#### 2.5.5. In Vitro Release Study

We conducted in vitro methotrexate release tests using dialysis [37,38] under different pH environments (pH 1.2 (HCl/NaCl solution) and 7.4 (PBS solution)). In order to predict the drug release pattern in gastric fluid and general in vivo environment following oral and intravenous (IV) IV administration of formulation or drug, the in vitro environment was set to pH 1.2 and 7.4. In other words, pH 1.2 and 7.4 environments set here potentially represent the gastric fluid and blood environments, respectively. Briefly, dialysis tubes (molecular weight cut-off 12 kDa; Sigma-Aldrich) containing 1.5 mL of methotrexate-loaded nanoemulsion and methotrexate solution were immersed into 10 mL of each pH medium and agitated at 37 °C and a rate of 50 opm in a water bath. Whole medium (10 mL) was withdrawn 5, 10, 20, 30, 45, and 75 min after incubation, and the same volume of fresh medium was immediately replaced. The amount of methotrexate released in each medium was measured using UPLC-MS/MS.

### 2.6. In Vivo Studies of Nanoemulsion

#### 2.6.1. Animal Experiments

Male Sprague–Dawley rats (7–9 weeks, 245–260 g) were purchased from Damul Science (Daejeon, Republic of Korea). Prior to experiments, all rats were housed under environmentally controlled temperature (23 ± 1 °C) and relative humidity (50 ± 5%). They were given a 12 h/12 h light/dark cycle, with food and water provided ad libitum. Animal experiments were approved by Chonnam National University Animal Experimental Ethics Committee, Republic of Korea (approval number: CNU IACUC-YB-2017-47, 6.7.2017). The procedures were conducted according to the revised Guidelines for Ethical Conduct in the Care and Use of Animals and the rules of Good Laboratory Practice. The rats were fasted overnight before drug administration with free access to water, and then they were randomly divided into one of four groups (*n* = 5 in each group) as follows: oral administration for free methotrexate or methotrexate-loaded nanoemulsion at a dose of 0.06 mg/kg methotrexate and IV administration for free methotrexate or methotrexate-loaded nanoemulsion at a dose of 0.024 mg/kg methotrexate. The dosage volume was 2 mL/kg for oral administration and 0.8 mL/kg for IV administration. Herein, the doses of drug administered to rats were set in consideration of the methotrexate concentration (0.03 mg/mL) in the nanoemulsion formulation optimized in this study. Free methotrexate or methotrexate-loaded nanoemulsion was administered using oral gavage, and IV administration was into the tail vein of lightly ether-anesthetized rats. Approximately 0.25-mL samples of blood were taken from the jugular vein at predetermined time points (0, 0.25, 0.5, 0.75, 1, 2, 4, 6, 8, and 12 h after administration) and then placed in heparinized microtubes (Axygen, Inc., Union City, CA, USA). After the blood samples were centrifuged at 10,000× *g* for 10 min, the separated plasma samples were stored at −80 °C until analysis.

In addition, rats were also randomly divided into to the experimental groups described above to evaluate the delivery of methotrexate to lymphatic system and tissues. After 2.5 h of drug administration, whole blood was drawn from the rat abdominal aorta. Then, the lymphatic system (axillary and mesenteric lymph nodes) and tissues (liver, kidney, spleen, and thymus) were collected. These samples were diluted with phosphate buffered saline at a 1/4 (*w*/*v*) ratio and immediately homogenized. The suspensions were stored at −80 °C until analysis.

#### 2.6.2. Quantification of Methotrexate in Biological Samples

We determined the methotrexate concentrations in the rat plasma, lymph nodes, and tissues using a validated UPLC-MS/MS method with reference to the analytical method in our previous study [25]. Briefly, methotrexate in biological samples was extracted using the solvents mixed with acetonitrile-ethyl acetate (9/1, *v*/*v*). Extracts were dried under nitrogen gas, and the dried residue was reconstituted in the mobile phase and injected into the LC-MS/MS system. Optimized chromatographic separation of methotrexate was conducted with a KINETEX core-shell C_18_ column (50 mm × 2.1 mm inner diameter, 1.7 μm particle size, Phenomenex, Torrance, CA, USA) at 40 °C. The mobile phase condition was 0.1% (*v*/*v*) formic acid in water (mobile phase A) and acetonitrile (mobile phase B) with a flow rate of 0.3 mL/min on the gradient control. Samples were quantified under positive electrospray ionization using multiple reaction monitoring modes at *m*/*z* 454.7→308.1 for methotrexate and *m*/*z* 180.0→110.1 for phenacetin (as an internal standard). The calibration curves obtained from all biological samples were in the range of 0.1–1000 ng/mL, with correlation coefficients (r^2^) > 0.99.

#### 2.6.3. Pharmacokinetic Analysis

Pharmacokinetic parameters were estimated by noncompartmental analysis using Phoenix WinNonlin™ software (version 8.2, Certara, Princeton, NJ, USA). Area under the plasma concentration-time curve (AUC) from 0 to t h (AUC_0-t_) was calculated by the linear trapezoidal rule. AUC from 0 to infinity (AUC_0–∞_) was calculated as AUC_0–t_ + C_t_/k (C_t_ was the last measured concentration, and k was the elimination rate constant in terminal phase). The maximum plasma concentration (C_max_) and the time to reach C_max_ (T_max_) were determined based on the plasma concentration-time curve. Half-life (t_1/2_) was calculated by the formula 0.693/k. Clearance (CL) and volume of distribution (V_d_) were defined as dose/AUC_0–∞, IV_ and dose/k·AUC_0–∞, IV_, respectively. Absolute oral bioavailability (F) for each formulation was calculated as (dose_IV_·AUC_0–∞, oral_)/(dose_oral_·AUC_0–∞, IV_) × 100. Targeting efficiencies of methotrexate to the lymphatic system and tissues were calculated as the ratio of the concentration of methotrexate in lymph nodes or tissues to plasma at 2.5 h after administration in each group. All data are expressed as mean ± standard deviation (SD).

### 2.7. Statistical Analysis

Statistical significance was analyzed using Student’s *t* test with significance at *p* < 0.05. The Statistical Package for the Social Sciences (SPSS) software (version 25, IBM, Armonk, NY, USA) was used for statistical analyses.

## 3. Results and Discussion

### 3.1. Screening of Solubility 

**Figure 1:**
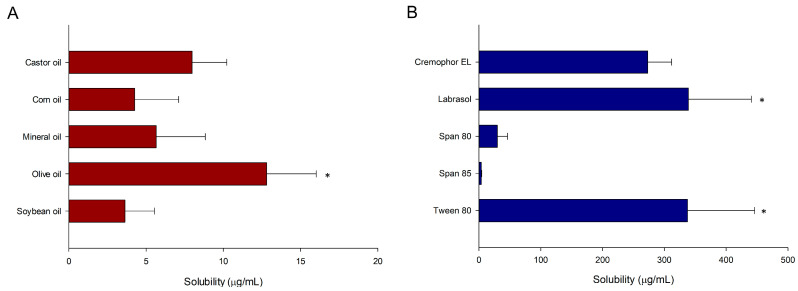
Solubility (at 25 °C) of methotrexate in various oils (**A**) and surfactants (**B**). Data are presented as mean ± SD (*n* = 3). * *p* < 0.05.

To develop an optimal nanoemulsion, it is necessary to identify components with high solubility for methotrexate. Excellent drug solubility in the oils and surfactants that constitute the nanoemulsion can increase the drug loading capacity in the formulation, thereby improving the drug bioavailability and target deliver. For this study, we selected the candidate oils and surfactants from among substances widely used in pharmaceutical formulations including nanoemulsions [39,40]. The solubility (at 25 °C) for methotrexate in various vehicles is presented in Figure 1. We observed the highest solubility in olive oil (12.81 ± 3.21 μg/mL, *p* < 0.05), and therefore, we determined the oil phase to be olive oil. Among the tested surfactants, Tween 80 and Labrasol showed higher solubility (337.61 ± 108.58 and 339.04 ± 101.81 μg/mL, respectively) than other surfactants, and there was no significant difference between the two (*p* > 0.05). Based on these results, we selected Tween 80 and Labrasol as candidate surfactants.

As already mentioned above (Section 2.2. Screening of Solubility), we used ethanol as a co-surfactant in this study. This is because ethanol as a co-surfactant can reduce the interfacial tension and provide a flexible interfacial layer [34,41]. Several previous researchers used ethanol as a co-surfactant for nanoemulsion [42,43]. In particular, to prepare methotrexate nanoemulsion, there was also a study on using it as an co-surfactant after the solubility of ethanol for methotrexate was tested [35].

### 3.2. Pseudo-Ternary Phase Diagrams

**Figure 2:**
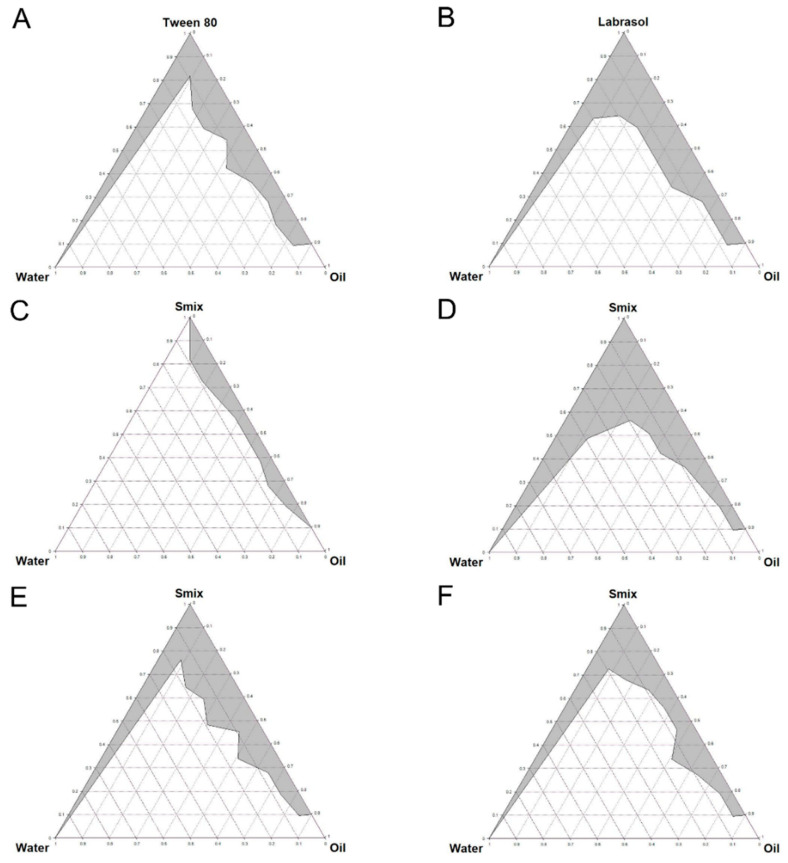
Pseudo-ternary phase diagrams composed of water, oil (olive oil) and surfactant. (**A**) Tween 80; (**B**) Labrasol; (**C**) Labrasol/ethanol (0.5/1, *v*/*v*); (**D**) Labrasol/ethanol (1/1, *v*/*v*); (**E**) Labrasol/ethanol (2/1, *v*/*v*); (**F**) Labrasol/ethanol (4/1, *v*/*v*).

Pseudo-ternary phase diagrams give information on the phase behaviors of mixtures with various component concentrations [13,36]. Based on the results of the solubility screening test, the pseudo-ternary phase diagrams were drawn using olive oil, water, and Tween 80 or Labrasol; Figure 2 depicts the diagram with water, olive oil and each surfactant, and the gray areas on the diagram represent nanoemulsion zone that have a transparent appearance. Figure 2A,B show the phase diagrams with Tween 80 and Labrasol used as surfactants, respectively. The area with Labrasol was broader, and, thus, we used it in the next step to study the effects of combination with co-surfactant. The phase diagrams with S_mix_ at different ratios (0.5:1, 1:1, 2:1, and 4:1, *v*/*v*) are shown in Figure 2C–F. We observed that the S_mix_ with the smallest surfactant ratio (Figure 2C) showed the smallest nanoemulsion area and that the largest area appeared when the ratio of surfactant and co-surfactant was 1:1 (*v*/*v*) (Figure 2D). The nanoemulsion area gradually decreased as the surfactant ratio increased thereafter. Therefore, we selected a 1:1 ratio (*v*/*v*) of S_mix_ as the optimal combination for a stable nanoemulsion.

### 3.3. Preparing the Nanoemulsion

As mentioned in the introduction, considering its properties and advantages, we judged the W/O/W nanoemulsion to be a suitable formulation for improving lymphatic drug delivery and bioavailability. In other words, W/O/W emulsions were expected to have better drug protection and degradation prevention by external environment than single (W/O or O/W) emulsions. However, in this regard, it seems necessary to conduct in-depth stability evaluation studies according to multiple emulsions and single emulsions in the future.

To prepare a suitable W/O/W nanoemulsion for methotrexate, we next explored the appropriate composition ratio of the formulation components. We also optimized the manufacturing process.

#### 3.3.1. Surfactant/Co-Surfactant Ratio in Water-in-Oil (W_1_/O) Emulsion

Based on the obtained pseudo-ternary phase diagram, S_mix_ is composed of surfactant (Labrasol) and co-surfactant (ethanol) at a ratio of 1:1 (*v*/*v*). To determine the optimal ratio of S_mix_ in emulsion, the effect of S_mix_ on droplet size was tested. S_mix_ ratio (*v*/*v*) in W_1_/O emulsion was varied 40, 50, and 60% while fixing the W_1_:O ratio as 1:4 (*v*/*v*). As shown in Table 1, the smallest W_1_/O emulsion (379.4 ± 21.43 nm) was produced when the proportion of S_mix_ was 60% (*v*/*v*). Moreover, on visual inspection, the emulsion appeared homogeneous, and no phase separation was observed for more than 24 h. The other two emulsions (40 and 50% S_mix_ ratio (*v*/*v*) in W_1_/O) were 18.1 and 13.2 times larger than the 60% S_mix_ ratio (*v*/*v*). We observed rapid phase separation within 5 min, which indicates the instability of these formulations; therefore, we determined the optimal ratio of S_mix_ in W_1_/O emulsion to be 60% (*v*/*v*) for a suitable W/O/W nanoemulsion. As the proportion of surfactant increases, it appears that the particle size decreases due to effective emulsification at the interface. However, an excessive amount of surfactant may cause formulation instability and in vivo toxic effects [44,45], so conditions of more than 60% S_mix_ (*v*/*v*) were excluded.

#### 3.3.2. Volume Ratio of Internal Water Phase to Oil Phase (W_1_:O) and W_1_/O Emulsion Phase to External Water Phase (W_1_/O:W_2_)

To investigate the droplet size depending on the ratio of oil and water phases (W_1_ and W_2_), the formulation was prepared by varying the phase volume ratio of W_1_:O and W_1_/O:W_2_. First, W/O/W emulsions were prepared by varying the W_1_:O ratio (*v*/*v*) as 10:90, 20:80, 30:70, and 40:60 while W_1_/O:W_2_ ratio (*v*/*v*) was arbitrarily fixed at 1:3. The S_mix_ ratio (*v*/*v*) in W_1_/O emulsion was set to 60%, which was optimized in the previous test (mentioned in 3.3.1. Surfactant/co-surfactant ratio in water-in-oil (W_1_/O) emulsion). Table 2 shows that the smallest nanoemulsion, 449.27 ± 31.68 nm, was produced with a W_1_:O ratio of 20:80 (*v*/*v*). The differences in droplet size were significant between the various volume ratios (*p* < 0.05), and from the results, we selected 20:80 (*v*/*v*) as the optimal ratio.

Second, we measured the nanoemulsion size changes with the different W_1_/O:W_2_ ratios when W_1_:O was fixed at 20:80 (*v*/*v*) and the S_mix_ ratio (*v*/*v*) in W_1_/O emulsion was also set to 60% as optimized in the previous test (3.3.1. Surfactant/co-surfactant ratio in water-in-oil (W_1_/O) emulsion). Among the three W_1_/O:W_2_ volume ratios (*v*/*v*), 1:3 showed the smallest emulsion (449.27 ± 31.68 nm; shown in Table 3). The results suggested that the 1:3 ratio (*v*/*v*) of W_1_/O:W_2_ was suitable for W/O/W nanoemulsion. Finally, we determined the optimal component composition for the W_1_/O/W_2_ emulsion as 15% (*v*/*v*) for S_mix_, 2% (*v*/*v*) for W_1_, 8% (*v*/*v*) for O, and 75% (*v*/*v*) for W_2_.

#### 3.3.3. Ratio of Methotrexate:Oil in Nanoemulsion

To determine the optimal ratio of methotrexate that can be enclosed in nanoemulsion, the size change was confirmed by varying the ratios of methotrexate and oil phase to 0:1, 1:4, 1:2, 1:1, and 2:1 (*w*/*v*). The composition of W_1_/O/W_2_ emulsion was as follows: 15% (*v*/*v*) for S_mix_, 2% (*v*/*v*) for W_1_, 8% (*v*/*v*) for O, and 75% (*v*/*v*) for W_2_. As shown in Table 4, compared with the blank nanoemulsion (containing no methotrexate, 0:1, *w*/*v*), the remaining conditions containing drug all increased in size. This may be because as drug is encapsulated in the formulation, the internal phase of formulation increases, resulting in an increase in droplet size compared to the blank. The results show the same trend as the previous study [25]. In addition, when methotrexate was contained with a ratio of 1:2 (*w*/*v*), the droplet size was the smallest at significance of *p* < 0.05, and at other ratios, the size increased significantly. This might be related to the size change and instability of the nanoemulsion depending on the methotrexate ratio. Based on our results, ratio of methotrexate and oil was optimized to be 1:2 (*w*/*v*).

#### 3.3.4. Number of Microfluidizer Passes 

**Figure 3:**
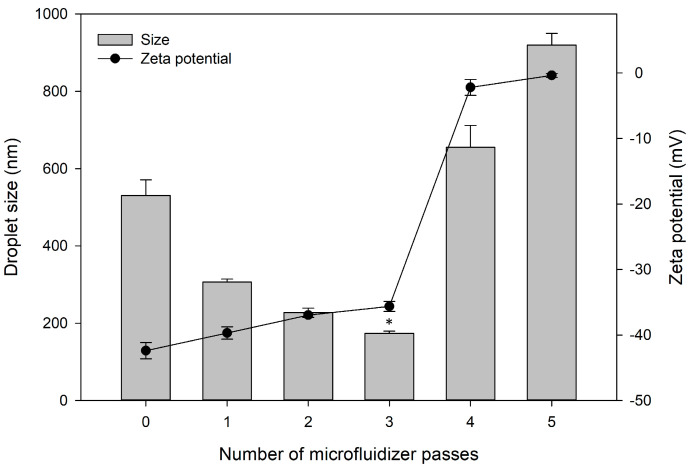
Effect of the number of microfluidizer passes on droplet size and zeta potential of methotrexate-loaded water-in-oil-in-water (W/O/W) nanoemulsion. Each value represents the mean and SD (*n* = 3, at 25 °C). * *p* < 0.05 in droplet size. The composition of W_1_/O/W_2_ emulsion consisted of 15% (*v*/*v*) for S_mix_, 2% (*v*/*v*) for W_1_, 8% (*v*/*v*) for O, and 75% (*v*/*v*) for W_2_. The ratio of methotrexate and oil was 1:2 (*w*/*v*).

In this study, we used a microfluidizer to reproducibly formulate a fine, homogeneous nano-sized emulsion. Microfluidization requires multiple passes to achieve a constant droplet size [46]. To evaluate the influence of the number of microfluidizer passes used to make the W_1_/O emulsion, the physicochemical properties of the double emulsion were measured by varying the number of passes from 0 to 5. Figure 3 shows the size and zeta potential of the methotrexate-loaded W/O/W nanoemulsions according to the number of passes. Reflecting the results of the optimization tests performed above, the composition of the W_1_/O/W_2_ emulsion was 15% (*v*/*v*) for S_mix_, 2% (*v*/*v*) for W_1_, 8% (*v*/*v*) for O, and 75% (*v*/*v*) for W_2_. The ratio of methotrexate and oil was 1:2 (*w*/*v*). As the number of passes increased from 0 to 3, the mean droplet size decreased from 530.53 ± 40.19 to 173.77 ± 5.76 nm. Moreover, the zeta potential had negative values under −30 mV (−42.40 ± 1.25 to −35.63 ± 0.78 mV), without much difference. In three microfluidizer passes, the smallest nanoemulsion was 173.77 ± 5.76 nm (*p* < 0.05). However, the emulsion sizes increased dramatically with four and five passes, and the zeta potentials increased close to neutral (−2.20 ± 1.21 and −0.37 ± 0.31 mV). This appears to have resulted in formulation instability or changes (in size and zeta potential) due to excessive physical stress.

The physicochemical properties of emulsion including droplet size and zeta potential are affected by the number of passes through a microfluidizer, which gives high shear stress to droplets. In our study, the droplet size decreased in up to three passes. This is because the repeated passes through the microfluidizer increase the probability that each droplet will experience high pressure [47]. However, the size increased rapidly, and its zeta potential also changed rapidly after exceeding three passes. Zeta potential indicates physical stability and electrostatic interactions between particles [35,48]. Emulsions with high zeta potential of greater 30 (negative or positive) are considered to be electrically stable, whereas emulsions with a low zeta potential tend to coagulate or flocculate, possibly resulting in poor physical stability [49]. Excessive shear of the microfluidizer appeared to cause the breakdown and instability of the formulation in four and five passes. Based on these findings, we concluded that three passes of the microfluidic cycle best stabilized the nanoemulsion of the composition and ratio we selected.

### 3.4. In Vitro Characterization

#### 3.4.1. Physicochemical Characteristics

We next evaluated the selected nanoemulsion for droplet size, zeta potential, EE, and pH. The final nanoemulsion consisted of 15% (*v*/*v*) for S_mix_, 2% (*v*/*v*) for W_1_, 8% (*v*/*v*) for O, 75% (*v*/*v*) for W_2_, 1:2 ratio (*w*/*v*) of methotrexate:oil, and three microfluidizer passes (all based on the optimization test results).

The nanoemulsion containing methotrexate was 173.77 ± 5.76 nm in size. In addition, the polydispersity index value was 0.10, and a uniform size distribution was shown. The proper size of nanoemulsion is an important factor because tiny particles (below 10 nm) can be eliminated by renal filtration, while large particles (above 200 nm) can be easily recognized by the reticuloendothelial system [50,51]. In other words, the size of methotrexate-loaded nanoemulsion we prepared in this study was within the target range. As mentioned above, emulsions primarily require zeta potential higher than ±30 mV, which ensures the physical stability. The zeta potential of the nanoemulsion we prepared was −35.63 ± 0.78 mV, indicating a stable formulation. The EE and pH of prepared nanoemulsion were 90.37 ± 0.96% and 4.07 ± 0.03, respectively; the pH of the formulation may be attributed to the weakly acidic nature of methotrexate. According to previous studies [52,53], the suitable pH range for both oral and IV administration was 2–9. Therefore, the pH of methotrexate-loaded nanoemulsions prepared in this study was judged to be suitable for both oral and IV administration.

We assessed the stability of the nanoemulsion formulation based on the changes in size, zeta potential, EE, and pH after storage at room temperature under shading conditions after two weeks. Compared with the properties of nanoemulsion immediately after formulation (0 day), there were no significant differences (*p* > 0.05) in size, zeta potential, EE, and pH at 1, 3, 7, and 14 days after preparation (Table 5). These results indicate that the methotrexate-loaded nanoemulsion was stable over 14 days.

#### 3.4.2. Morphology

**Figure 4:**
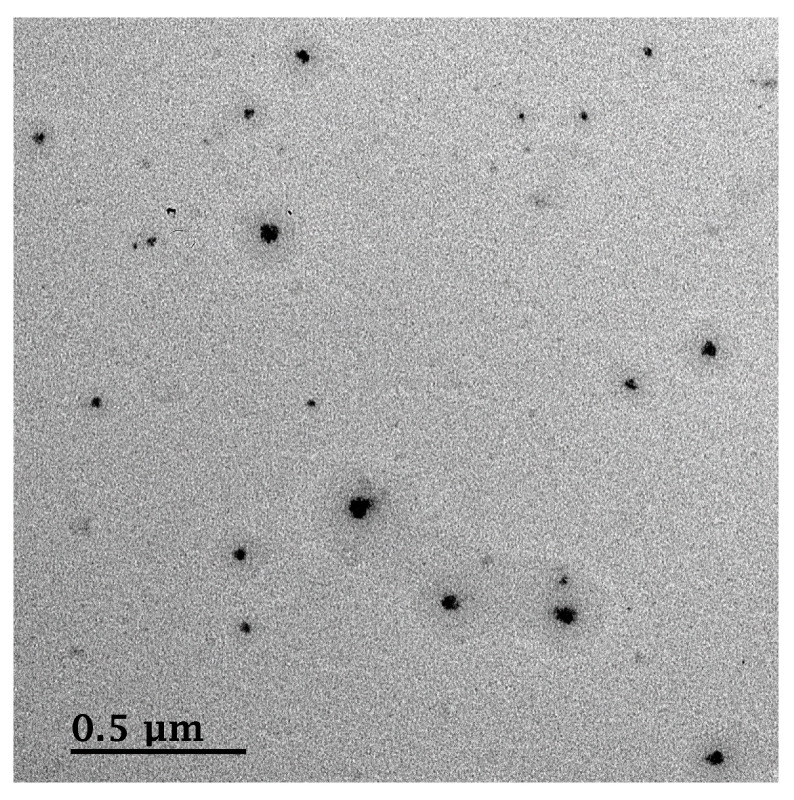
Field-emission transmission electron microscope (FE-TEM) image of methotrexate-loaded nanoemulsion (15% (*v*/*v*) for S_mix_, 2% (*v*/*v*) for W_1_, 8% (*v*/*v*) for O, 75% (*v*/*v*) for W_2_, 1:2 ratio (*w*/*v*) of methotrexate:oil, and three microfluidizer passes).

The morphological features of the finally optimized methotrexate-loaded nanoemulsion obtained using FE-TEM are shown in Figure 4. The conditions of the finally prepared formulation are as follows: (1) 15% (*v*/*v*) for S_mix_, 2% (*v*/*v*) for W_1_, 8% (*v*/*v*) for O, 75% (*v*/*v*) for W_1_ in W/O/W_2_ nanoemulsion (2) 1:2 ratio (*w*/*v*) of methotrexate:oil, and (3) three microfluidizer passes. The formulation was spherical, and the droplet size seen on FE-TEM is in agreement with the result of DLS measurement.

#### 3.4.3. In Vitro Drug Release

**Figure 5:**
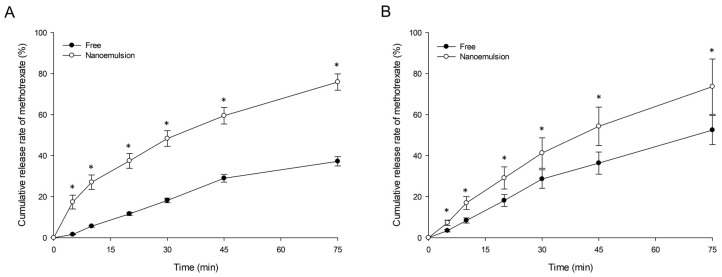
In vitro release profiles of free methotrexate and methotrexate-loaded nanoemulsion in pH 1.2 (**A**) and pH 7.4 (**B**) medium. Values are presented as the mean ± standard deviation (*n* = 3). * *p* < 0.05 compared with free methotrexate.

We investigated the initial in vitro release pattern of the nanoemulsion and free methotrexate using dialysis membrane filtration. Herein, the prepared nanoemulsion consisted of 15% (*v*/*v*) for S_mix_, 2% (*v*/*v*) for W_1_, 8% (*v*/*v*) for O, 75% (*v*/*v*) for W_2_, 1:2 ratio (*w*/*v*) of methotrexate:oil, and three microfluidizer passes. Taking into account the molecular weight of the drug and the characteristics of the emulsion type, we expected the release to occur within a short period of time, and thus, this short period was our focus. In the previously reported studies [13,54], drug release from formulations, such as emulsions, proceeded quickly within a relatively short time (about 60 min). Therefore, in this study, in vitro release test was set up to 75 min after incubation by referring to these previous studies. In Figure 5, in vitro release of methotrexate from the nanoemulsion was faster than that of free drug in both pH environments. The cumulative degrees of released methotrexate from the nanoemulsion for 75 min were 1.4 times (pH 7.4) and 2 times (pH 1.2) higher than the control. In addition, the drug release rate in the nanoemulsion was significantly higher at all time points; we attributed this result to the properties of nanoemulsion. Nanoemulsions have nano-sized small droplets, large surface area, and high interfacial area required for dissolution [35,55]. Therefore, the small droplet size and large surface area due to the structure or physical properties of nanoemulsions could cause rapid drug release in the media and passage through the dialysis membrane (by further reducing the free energy). In addition, it is thought that the surfactant contained in the formulation could have influenced the permeability of dialysis membrane. The in vitro release pattern (rapid drug release from the formulation) observed in our study tended to be consistent with previous related studies [13,55].

Additionally, more free methotrexate was released at pH 7.4 than at pH 1.2 until the last 75 min. This is because methotrexate is a weak acid, and thus, it is more soluble in a neutral environment than in an acidic environment. In case of nanoemulsion in contrast, there was no significant difference in the release rate by pH environment until the final time. This was likely because the drug was protected from the external environment by being encapsulated in the nanoemulsion.

### 3.5. In Vivo Studies

#### 3.5.1. Quantification of Methotrexate in Biological Samples

As mentioned in Section 2.6.2. Determining Methotrexate in Biological Samples, we quantified the methotrexate concentrations in rat biological samples following our previously conducted method [25]. We thoroughly validated UPLC-MS/MS as a method for determining the concentrations for linearity, precision, accuracy, stability, recovery, and matrix effect following Food and Drug Administration validation guidance [56]. The relevant contents were presented in detail in our previous report [25]. The analytical method we used to quantify methotrexate in rat samples was well applied in this study.

#### 3.5.2. Pharmacokinetics and Targeting Delivery Study

In the two rat groups that received oral administration of methotrexate (free and as methotrexate-loaded nanoemulsion), we observed no significant side effects or lethality within at least 24 h after administration. In contrast, in our preliminary study, we observed 60% mortality and hematuria in rats when a volume of 1.5 mL/kg of methotrexate-loaded nanoemulsion was administered intravenously; moreover, lethality occurred within 5 min after administration of the formulation. It appeared that a relatively high amount of surfactant in formulation was injected into blood vessels to cause hemolysis [57]. Accordingly, when we applied a lower volume of 0.8 mL/kg in IV administration, we observed no side effects including lethality or hematuria.

**Figure 6:**
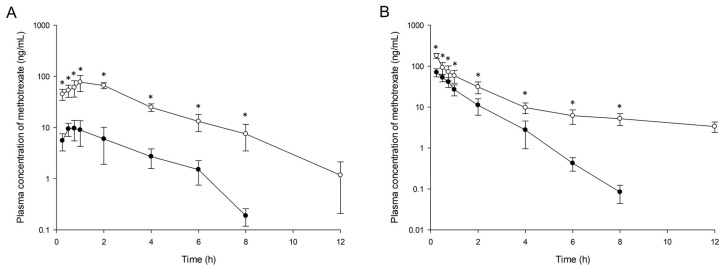
Plasma concentration profiles of methotrexate in rats after oral (**A**) and intravenous (**B**) administration of free methotrexate (-●-) or methotrexate-loaded nanoemulsion (-○-). Data are presented as the mean ± standard deviation (*n* = 5). * *p* < 0.05 compared with free methotrexate.

We conducted pharmacokinetic analysis of methotrexate after oral or IV administration of free methotrexate or methotrexate-loaded nanoemulsion to rats. Plasma concentration-time profiles of methotrexate are presented in Figure 6. Compared with the control (free methotrexate), the nanoemulsion formulation contributed to significantly (*p* < 0.05) higher plasma methotrexate concentrations over the whole time period, and the drug remained in plasma for a longer period in both oral and IV administration. When nanoemulsion was administered, methotrexate was detected in rat plasma up to 12 h after both oral and IV administration, whereas free methotrexate was detected only up to 8 h after administration. Methotrexate was eliminated from the nanoemulsion more slowly in the plasma than was the free drug.

Table 6 lists the pharmacokinetic parameters of methotrexate estimated by noncompartmental analysis. The AUC, C_max_, t_1/2_, and V_d_ of methotrexate from nanoemulsion were significantly greater than those of control, with lower CL (*p* < 0.05). This result is also consistent with the pharmacokinetic finding of the slower elimination of methotrexate in nanoemulsion. In contrast, there were no significant differences in T_max_ after oral administration between the two formulations, indicating that there is no significant difference in the methotrexate absorption rate in the gastrointestinal tract between free drug and nanoemulsion. However, when comparing the C_max_ between formulations, the degree of drug absorption in the nanoemulsion was greater than that of free drug. In addition, nanoemulsion increased the F of methotrexate from 12.71 to 38.77%, about three times higher than in the control group. In our results, nanoemulsion improved the bioavailability of methotrexate and enabled its long retention in the blood.

**Figure 7:**
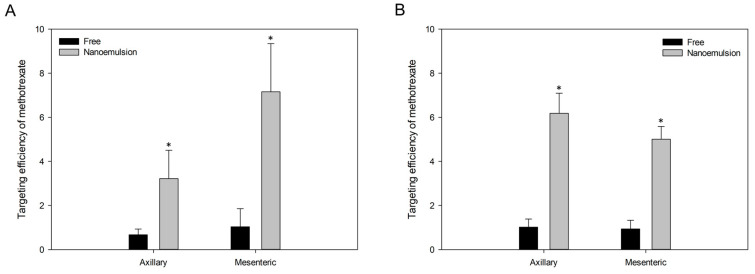
Lymphatic targeting efficiency of methotrexate to axillary and mesenteric lymph nodes at 2.5 h after oral (**A**) or intravenous (**B**) administration of free methotrexate and methotrexate-loaded nanoemulsion to rats. Vertical bars represent standard deviation of the mean (*n* = 5). * *p* < 0.05 compared with free methotrexate.

To evaluate the improvement in lymphatic delivery of the formulation, we compared lymphatic targeting efficiency by calculating the ratio of the concentration in lymph nodes to the concentration in plasma. Overall, drug concentration and targeting efficiency in axillary and mesenteric lymph nodes were significantly higher (*p* < 0.05) with methotrexate-loaded nanoemulsion in both oral and IV routes (Figure 7). The nanoemulsion enhanced lymphatic targeting efficiency of methotrexate from 4.8 to 6.9 times. The results showed that nanoemulsion more effectively transported methotrexate to the lymphatic tissue, and we expect that this increased lymphatic target delivery would allow for lower drug doses in clinical use. The size characteristics of nanoemulsions might be related to the enhanced lymphatic delivery of methotrexate in both oral and IV routes. In other words, the nano-sized formulation would have increased passive diffusion and delivery of drug to lymphatic tissue. In addition, oral administration of nanoemulsion showed a stronger rate of delivery to the mesenteric lymph node than to the axillary node, whereas the targeting after IV administration was greater to the axillary lymph node. We consider that this difference occurred because of the difference in distribution caused by the different administration routes. Moreover, Labrasol, a surfactant that enhances intestinal membrane permeability, might have helped to increase the absorption of drug in the gastrointestinal tract [58,59].

**Figure 8:**
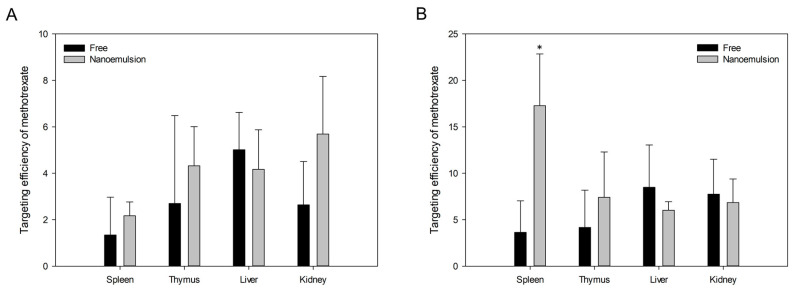
Targeting efficiency of methotrexate to spleen, thymus, liver and kidney at 2.5 h after oral (**A**) or intravenous (**B**) administration of free methotrexate and methotrexate-loaded nanoemulsion to rats. Each value represents the mean and standard deviation (*n* = 5). * *p* < 0.05 compared with free methotrexate.

To confirm the distribution in tissues rather than in the lymphatic system, we quantified the methotrexate in four rat organs: the spleen and thymus, which are immune-related organs, and the liver and kidney, which are known as the sites of major methotrexate side effects. We calculated targeting efficiency for each organ using the same method we used to measure lymphatic targeting efficiency; Figure 8 shows the methotrexate targeting efficiencies in the spleen, thymus, liver, and kidney. Contrary to the results in lymph nodes, there were no distinct differences in targeting delivery to most tissues between nanoemulsion and free drug. When the prepared emulsion was administered intravenously, the targeting efficiency in the spleen was higher, but the efficiency to the liver, kidney, and thymus was not significantly different from that in the control group with both oral and IV administration. In particular, we confirmed that compared with free drug, the nanoemulsion did not distribute significantly more methotrexate to the liver and kidney, which are organs related to major methotrexate side effects, while increasing the transition to lymph nodes.

To summarize the results of tissue distribution study, we confirmed that nanoemulsion was a formulation that could significantly improve lymphatic delivery without significantly greater side effects of methotrexate than free methotrexate at the same drug dose. This suggests the possibility of improving the therapeutic effect of methotrexate, which is widely used for immune-related diseases in addition to anticancer treatment, by delivering more methotrexate to the immune system than conventional drug through nanoemulsification. Based on this pharmacokinetic study, it is necessary to study the efficacy of methotrexate-loaded nanoemulsions for disease models in the future. Moreover, it can be expanded to clinical studies of methotrexate-loaded nanoemulsions.

## 4. Conclusions

In this study, we prepared nanoemulsion as a drug delivery system to improve lymphatic delivery of methotrexate. In consideration of the solubility of methotrexate, we selected olive oil and Labrasol as the appropriate oil and surfactant for the nanoemulsion. We optimized the preparation process and conditions using microfluidization to prepare an optimal nano-sized W/O/W emulsion. By evaluating the physicochemical properties, we confirmed that the prepared nanoemulsion was in a stable, uniform nano-sized formulation. Additionally, an in vitro release test showed that methotrexate-loaded nanoemulsion released the drug more rapidly than the free drug was released. From in vivo studies, we observed significantly elevated C_max_, AUC, half-life, and bioavailability in both oral and IV administration of nanoemulsion, and the targeting efficiency to lymph nodes also significantly improved. We conclude that the nanoemulsion we prepared in this study could be a good candidate formulation for improving lymphatic transport of drugs. Moreover, it might also contribute to lowering clinical drug doses and, thus, reducing side effects.

## Figures and Tables

**Table 1:** Effect of S_mix_ ratio (*v*/*v*) on the size of W_1_/O emulsion (at 25 °C, *n* = 3, mean ± SD).

S_mix_ (%, *v*/*v*)	W_1_/O (%, *v*/*v*)	Size (nm)
**40**	12/48	6865.13 ± 438.91
**50**	10/40	5018.33 ± 52.21
**60 ^†^**	8/32	379.4 ± 21.43

S_mix_ means surfactant/co-surfactant mixture. ^†^ Selected.

**Table 2:** Effect of W_1_:O ratio (*v*/*v*) on droplet size of double emulsion (at 25 °C, *n* = 3, mean ± SD).

W_1_:O Ratio (*v*/*v*)	Size (nm)
**10:90**	1130.73 ± 112.04
**20:80 ^†^**	449.27 ± 31.68
**30:70**	586.73 ± 20.87
**40:60**	5035.20 ± 579.16

S_mix_ ratio in W_1_/O emulsion was fixed as 60% (*v*/*v*). W_1_/O:W_2_ ratio was fixed as 1:3 (*v*/*v*). ^†^ Selected.

**Table 3:** Effect of W_1_/O:W_2_ ratio (*v*/*v*) on droplet size of double emulsion (at 25 °C, *n* = 3, mean ± SD).

W_1_/O:W_2_ Ratio (*v*/*v*)	Size (nm)
**1:2**	1142.17 ± 102.62
**1:3 ^†^**	449.27 ± 31.68
**1:4**	1812.30 ± 232.11

S_mix_ ratio in W_1_/O emulsion was fixed as 60% (*v*/*v*), W_1_/O ratio was fixed as 20:80 (*v*/*v*), ^†^ Selected.

**Table 4:** Effect of methotrexate/oil ratio (*w*/*v*) on droplet size of double emulsion (at 25 °C, *n* = 3, mean ± SD).

Methotrexate/Oil Ratio (*w*/*v*)	Size (nm)
**0:1**	449.27 ± 31.68
**1:4**	1142.00 ± 117.55
**1:2 ^†^**	530.53 ± 40.19
**1:1**	713.43 ± 43.75
**2:1**	2206.67 ± 249.35

The composition of W_1_/O/W_2_ emulsion consisted of 15% (*v*/*v*) for S_mix_, 2% (*v*/*v*) for W_1_, 8% (*v*/*v*) for O, and 75% (*v/v*) for W_2_. ^†^ Selected.

**Table 5:** Stability profiles on physicochemical characteristics of nanoemulsion (at 25 °C, *n* = 3, mean ± SD).

Time (Day)	Size (nm)	Zeta Potential (mV)	Encapsulation Efficiency (%)	pH
**0**	173.77 ± 5.76	−35.63 ± 0.78	90.37 ± 0.96	4.07 ± 0.03
**1**	178.23 ± 6.95	−35.57 ± 0.65	90.20 ± 1.21	4.08 ± 0.06
**3**	178.83 ± 9.14	−35.43 ± 0.78	90.77 ± 1.12	4.11 ± 0.05
**7**	178.47 ± 4.96	−35.23 ± 0.80	89.57 ± 0.47	4.06 ± 0.05
**14**	178.57 ± 5.73	−34.93 ± 0.65	90.13 ± 1.46	4.09 ± 0.06

The condition of W_1_/O/W_2_ emulsion consisted of 15% (*v*/*v*) for S_mix_, 2% (*v*/*v*) for W_1_, 8% (*v*/*v*) for O, 75% (*v*/*v*) for W_2_, 1:2 ratio (*w*/*v*) of methotrexate:oil, and three microfluidizer passes.

**Table 6:** Pharmacokinetic parameters of methotrexate in rats after oral or intravenous administration of free methotrexate and methotrexate-loaded nanoemulsion (*n* = 5, mean ± SD).

Parameters	Oral (0.06 mg/kg as Methotrexate)		IV (0.024 mg/kg as Methotrexate)	
	Free	Nanoemulsion	Free	Nanoemulsion
AUC_0–t_ (ng·h/mL)	29.31 ± 7.70	288.35 ± 51.14 *	93.10 ± 17.72	268.94 ± 41.85 *
AUC_0–∞_ (ng·h/mL)	29.62 ± 7.77	291.34 ± 54.01 *	93.20 ± 17.70	300.56 ± 36.10 *
C_max_ (ng/mL)	12.13 ± 3.38	81.72 ± 23.01 *	-	-
T_max_ (h)	0.70 ± 0.21	1.35 ± 0.60	-	-
t_1/2_ (h)	1.11 ± 0.20	1.58 ± 0.30 *	0.83 ± 0.15	6.38 ± 1.77 *
CL (mL/h·kg)	-	-	263.98 ± 43.02	80.94 ± 11.38 *
V_d_ (mL/kg)	-	-	320.49 ± 88.50	748.38 ± 232.69 *
F (%)	12.71	38.77	-	-

* *p* < 0.05 between free methotrexate and nanoemulsion groups.
